# Glycosylated Notch and Cancer

**DOI:** 10.3389/fonc.2016.00037

**Published:** 2016-02-18

**Authors:** Shanmugasundaram Pakkiriswami, Africa Couto, Usha Nagarajan, Marios Georgiou

**Affiliations:** ^1^Department of Biology, University of Western Ontario, London, ON, Canada; ^2^School of Life Sciences, Queen’s Medical Centre, University of Nottingham, Nottingham, UK; ^3^School of Chemical and Biotechnology, Shanmugha Arts, Science, Technology & Research Academy, Thanjavur, India

**Keywords:** glycosyltransferases, Notch signaling, tumorigenesis, epidermal growth factor

## Abstract

Glycosylation is one of the key components influencing several signaling pathways implicated in cell survival and growth. The Notch signaling pathway plays a pivotal role in numerous cell fate specifications during metazoan development. Both Notch and its ligands are repeatedly glycosylated by the addition of sugar moieties, such as O-fucose, O-glucose, or O-xylose, to bring about structural and functional changes. Disruption to glycosylation processes of Notch proteins result in developmental disorders and disease, including cancer. This review summarizes the importance and recent updates on the role of glycosylated Notch proteins in tumorigenesis and tumor metastasis.

## Introduction

It has been 60 years since the discovery of a link between the changes in protein glycosylation and oncogenic transformation (1). Numerous studies in cancer biology have supported this finding and underscored the significance of glycosylation in tumorigenesis and metastasis. Numerous proteins, such as mucins, selectins, gonadrotrophins, with altered glycosylation have been implicated in tumorigenesis (2, 3). Extensive research has been carried out to understand the glycobiology of the cancerous cell (4, 5). Cancer is a complex disease requiring several accumulated mutations, whose progression additionally depends on tumor cell interactions with the surrounding environment (6). Cell-surface proteins and changes associated with them define the course of cell-to-cell interactions. Altered glycosylated cell-surface proteins are one of the unique features of a cancerous cell (7), and specific glycan changes are critical during tumorigenesis and metastasis (8, 9). Several altered glycans serve as biomarkers to identify malignant cells undergoing epithelial–mesenchymal transition (EMT) and metastasis (9–11). Many essential glycosylated proteins, such as Notch, are altered in malignant cells, and the recent finding of GALNT11 as a new molecular marker in Notch-mediated chronic lymphocyte leukemia (CLL) (12) has increased interest in understanding Notch glycosylation.

The Notch signaling pathway facilitates short-range cell–cell communication to play a central role in proliferation and differentiation during animal development (13, 14). Notch signaling regulates a plethora of genes implicated in various cellular processes, and its signaling activity is extremely sensitive to the Notch receptor levels. Therefore, any slight modulation in Notch activity can perturb the regulation of gene expression and thus promoting several disorders, including cancer. Intriguingly, aberrant Notch signaling activity is highly implicated in several forms of leukemia and solid tumor development (11). T-cell acute lymphoblastic (T-ALL) neoplasm is one of the earliest diseases to be associated with the Notch signaling pathway (15). Membrane-bound Notch proteins (Notch receptor and its ligands) undergo rigorous glycosylation to accomplish its activity. Interestingly, deregulation of the components involved in glycosylating Notch proteins are implicated in Notch-induced tumorigenesis (16–19).

There is extremely robust evidence to suggest that aberrant Notch activity (11, 20, 21) or changes in glycosylation (5, 22, 23) can promote EMT and tumor development, despite the unclear role of glycosylated Notch proteins in relation to tumorigenesis. This review discusses recent advances in our understanding of glycosylation of Notch proteins and the impact of altered Notch glycans in promoting tumorigenesis and metastasis.

## An Overview of Notch Signaling Pathway

A century ago, flies with Notch-ed wing phenotype led to identification (24) and characterization of evolutionarily conserved Notch signaling pathway (25, 26). The signaling pathway comprising Notch receptor and Delta/Serrate/LAG-2 (DSL) family of ligands play crucial role in determining cell-fate choices in all animals (13, 27). While *Drosophila* has one Notch receptor (28), mammalians have four homologs (Notch1–4) (29) with an extracellular domain (ECD) and an intracellular domain (ICD). Both Notch receptor and DSL ligands show a high degree of structural similarities in the ECD (30).

**Figure 1:**
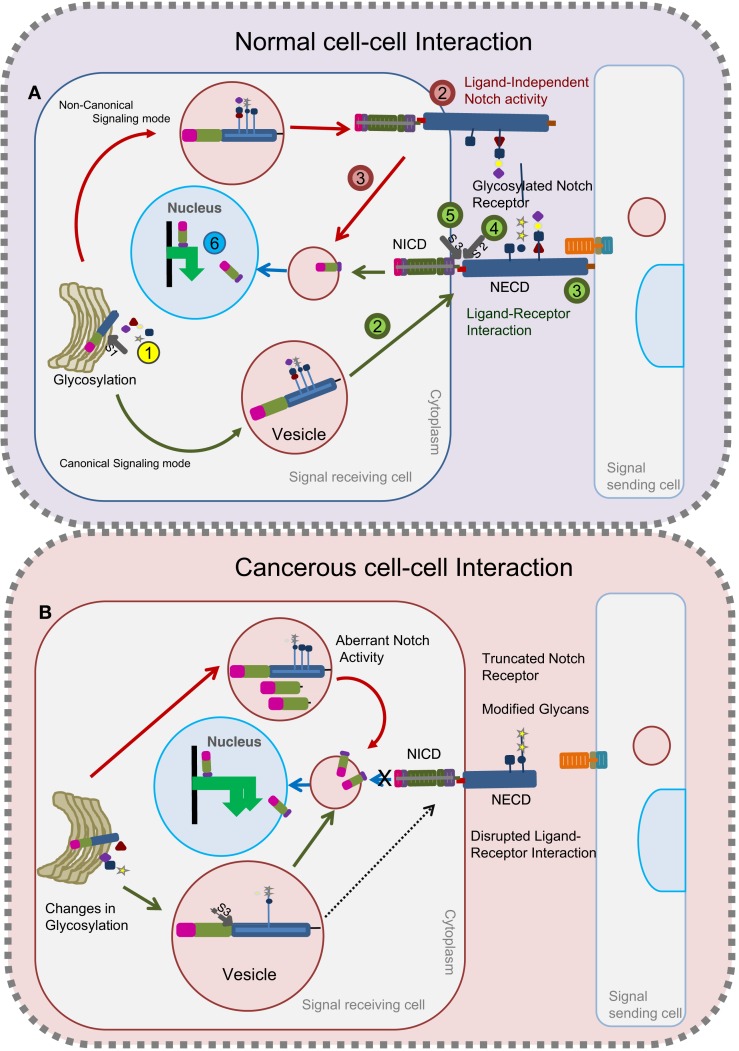
**Glycosylated Notch-mediated cell–cell interactions in normal cells and cancerous cells**. In normal cells **(A)**, glycosylation on the Notch receptor takes place in the Golgi bodies followed by proteolytic (S1) furin cleavage (Step 1, yellow). Depending on the glycosylation cues, glycosylated Notch receptor takes either a canonical route (as shown by green arrows) or a non-canonical route (as shown by red arrows) to release the NICD fragment. Through the canonical mode (green) of a signal-receiving cell, glycosylated Notch receptor is transferred to the plasma membrane (Step 2) to interact with the ligands on the signal-sending cell (Step 3). Following this, the Notch receptor undergoes two successive proteolytic cleavages (S2) (Step 4) and (S3) (Step 5) to release the NICD that translocates into the nucleus to activate target genes (Step 6). In the non-canonical route (red), the Notch receptor does not interact with ligands (Step 2) but gets proteolytically cleaved inside the vesicles to release the NICD fragment (Step 3) that translocates into the nucleus to activate the target genes (Step 6). In cancerous cells **(B)**, changes in glycosylation lead to the production of Notch receptors with modified glycans or truncated Notch receptors. Notch receptors with modified glycans undergo unusual S3 cleavage in the vesicles that releases NICD to activate Notch targets. Truncated Notch receptors and Notch receptors with modified glycans that reach the plasma membrane cannot interact with ligands on the signal-sending cell to release the NICD.

Notch signaling involves receptor activation, Notch ICD (NICD) generation, and target stimulation (Figure 1A). Nascent Notch protein is initially glycosylated in the ER and Golgi apparatus (17, 31–33). In mammals, it is proteolytically cleaved by Furin at site 1 (S1) (this cleavage does not occur in *Drosophila*) (34). Following this, the mature Notch receptor heterodimer, comprising the ECD and transmembrane-ICD, gets tethered to the cell surface of the signal-receiving cell. Notch receptor interaction with membrane-bound ligands such as Delta/Serrate/LAG-2 (DSL) family proteins in the signal-sending cell initiates two successive proteolytic cleavages at site 2 (S2) and site 3 (S3) mediated by a disintegrin and metalloprotease (ADAM) and presenilin/γ-secretase complex, respectively. The NICD is then released, which translocates into the nucleus and binds to CSL (CBF1/SuH/LAG-1) transcriptional regulators to activate target genes (14, 35). The events that lead to the release of the Notch ICD (and Notch activation) rely on Notch ECD shedding. Evidences indicate presence of a non-canonical mode of signaling without ligand–receptor interaction to release NICD [Figure 1A (36, 37)]. Therefore, factors that influence shedding of the Notch ECD (either positively or negatively) can directly modulate Notch activity. Numerous extra- and intracellular modulators involving glycosylation, and trafficking machineries maintain the cellular pool of Notch in a context-specific manner.

Notch protein exerts its biological functions by both canonical (ligand-dependent) and non-canonical (ligand-independent) signaling modes (Figure 1A). Contradicting earlier reports that ligand-dependent Notch activity alone is indispensable for the positive regulation of Notch signaling, recent work suggests that ligand-independent Notch signaling also plays a crucial positive role in regulating Notch activity (36, 38). Namely, changes in trafficking can lead to ligand-independent signaling (Figure 1A). Defects in any one of the two signaling modes can lead to tumorigenesis and tumor progression. Strikingly, glycosylation is one such process that modulates ligand–receptor binding and trafficking activities [Figure 1B (39, 40)]. In the following section of this review, we discuss the impact of glycosylation on Notch and its ligands to accomplish its biological function.

## Glycosylation of Notch Proteins

Glycosylation is an enzymatic reaction that mediates a chemical linkage of mono- or polysaccharides (glycans) onto other saccharides, proteins, or lipids occurring in Golgi apparatus and endoplasmic reticulum (ER). Nascent Notch proteins enroute the secretory pathway (41) to undergo a rigorous glycosylation on their ECD with 29–36 epidermal growth factors (EGF)-like repeats to emerge as a mature receptor and get localized on the cell surface (42). Predominantly, Notch receptor undergoes O-glycosylation at serine/threonine residues (31), and to a lesser extent, *N*-glycosylation on AsnXSer/Thr residues of EGF repeats (17).

**Figure 2:**
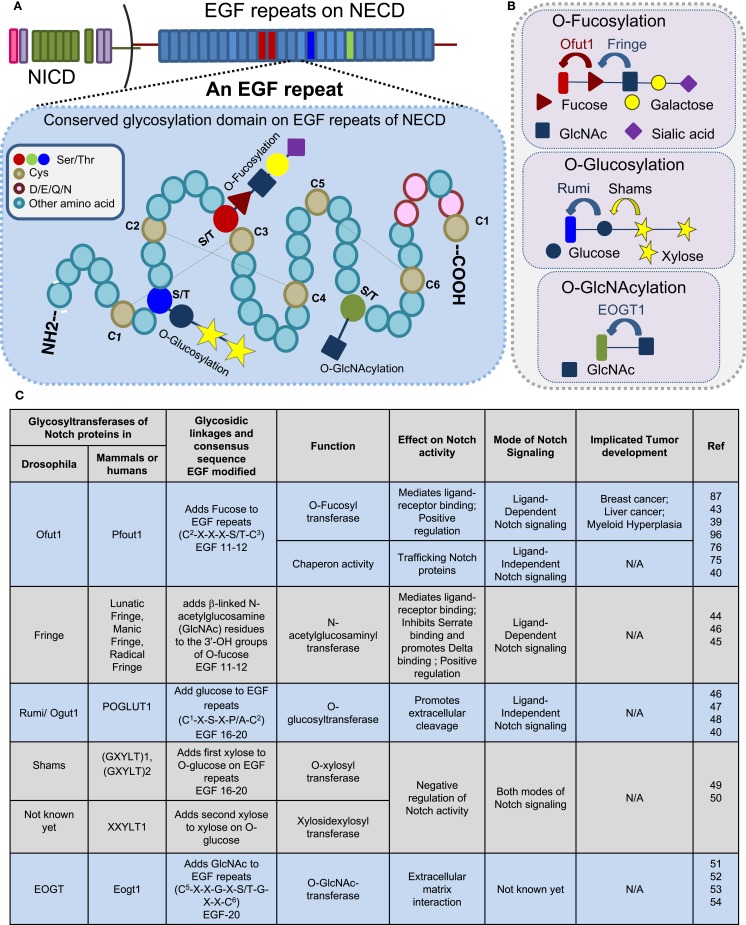
**Glycosylation sites present on EGF repeats of the Notch extracellular domain**. Consensus sites **(A)** and glycosyltransferases **(B)** of O-fucosylation, O-glycosylation, and O-GlcNAcylation that occur on an EGF repeat of NECD. **(C)** Table displaying a partial list of the glycosyltransferases that modify the Notch receptor.

The EGF repeats are modified by O-fucose, O-glucose, O-GlcNAc, and O-xylose (Figure 2). This short EGF repeat has six conserved Cys residues that form three disulfide bridges, wherein O-fucosylation at C^2^–X–X–X–S/T–C^3^ is mediated by O-fucosyltransferase 1 (O-FucT-1) (encoded by *Ofut1* gene in *Drosophila* and *Pofut1* in mammals) (39, 43) and elongated by Fringe, an *N*-acetylglucosaminyl transferase. Fucosylation is one of the prevalent glycosylation types on Notch proteins. Fringe is essential to promote Notch/Delta-binding, in preference to Notch/Serrate, whose interaction is inhibited by this modification (44, 45). Similarly, O-glycosylation (C^1^–X–S–X–P/A–C^2^) is mediated by O-glucosyltransferase, *Rumi* in *Drosophila* or POGLUT1 in mammals (40, 46–48), and elongated by Shams, a xylosyltransferase. In humans, xylosyltransferase (GXYLT)1 and (GXYLT)2, that add first and second xylose residues to Notch EGF repeats, have been identified (49, 50). Although Rumi is not required for the ligand-binding activity of Notch, it has been suggested that it functions to promote extracellular cleavage. In contrast to the glucose residues, xylosylation negatively regulates Notch signaling. A unique non-nucleocytoplasmic O-GlcNAc is reported to occur on the consensus sequence of Notch C^5^–X–X–G–X–S/T–G–X–X–C^6^ by EGF-specific O-GlcNAc-transferase (EOGT) in *Drosophila* and Eogt1 in mammals that mediate extracellular matrix interactions (51–54). The specific role of O-GlcNAc modifications on Notch activity is still not clear (Figure 2C). Recently, the presence of mucin-type-O-GalNAc glycans on the Notch ECD and N-glycans have been reported (19). The last four amino acids in the spacer between EGF repeats cooperates with calcium-ion binding and plays an important role in enhancing the rigidity and stability of EGF repeats (55). Recent research suggests that a strong crosstalk exists between glycosylating machinery and calcium modulating chaperones in regulating Notch activity.

## Notch-Induced Tumorigenesis

Recent glycomic studies suggest that common glycosylation changes associated with tumor development are fucosylation, sialylation, O-glycan truncation, and O- and N-linked branching (8), and most of these changes are frequently associated with Notch proteins. Notch pathway cooperates with other mutations or deregulation in other oncogenic or tumor-suppressive genes of other signaling pathways, polarity regulators, and endocytic compartments to potentiate tumor progression (56–58).

Aberrant Notch activity has distinct roles in the development of several solid and hematopoietic tumors, and it has been shown to have either oncogenic or tumor-suppressive roles in a context-specific manner (21). In hematopoietic cancers, for example, T cell active lymphoblastic leukemia (T-ALL), Notch has an oncogenic role (59, 60), while in acute myeloid leukemia (AML), it has a tumor-suppressive role (61, 62). However, in solid tumors, such as hepatocellular carcinoma (HCC) (63) and medulloblastoma (64), Notch may have either an oncogenic or tumor-suppressive role depending on context (65, 66). It has been suggested that the switch between canonical and non-canonical Notch signaling can have a tumor-suppressive role, as demonstrated with Notch1 in the mouse (67). Not only does the glycosylation process of the Notch receptor aid in ligand-dependent signaling activity, but also reports suggest that changes in glycosylation events may lead to ligand-independent Notch activation (37). Most importantly changes in glycosylation could switch from one mode of signaling to the other, leading to deregulated Notch activity.

### Ligand-Dependent Notch-Mediated Tumorigenesis

Notch protein undergoes extensive post-translational modification (68). To a larger extent, the ligand-dependent Notch signaling pathway requires glycosylation of the Notch ECD for signaling activation. It has been demonstrated that altered carbohydrate structure can play a very significant role in modulating ligand-binding activity (31, 69). Glycan modifications on EGF repeats of the Notch receptor indicate that the EGF8 repeat is required for Serrate-specific binding (70), while EGF12 is specifically required for Fringe function to inhibit Serrate and promote Delta-binding (16, 71), which mediates positive regulation on Notch signaling (Figure 2). Biochemical studies suggest that O-fucose addition on EGF14 leads to either dysregulated receptor–ligand activation or truncation effects. Fucosylation, which modulates the receptor–­ligand interaction, is an important epitope on the EGF repeat (72–74) and impairment to this process is implicated with various forms of malignancies. Reports indicate that aberrant ligand-dependent Notch activity is highly associated with development of HCC, T-cell leukemia, and breast cancer (23). Interestingly, in humans, upregulation and aberrant gene expression of α-1,6-fucosyltransferase encoded by FUT8 is associated with development of breast cancer (75) and liver cancer (76).

Intriguingly, deletion or truncation of EGF repeats that impair the ligand–receptor interaction has been implicated in tumorigenesis. Such truncation may emerge due to defects in the glycosyl transferases or other factors that cooperate with them to enhance the receptor–ligand interaction. In squamous cell lung carcinoma (SqCC), where the tumor-suppressive role of Notch is impaired, it has been demonstrated that loss of EGF repeats generates truncated receptors that disrupt ligand-binding activity (Figure 1B) (77). Defects in Notch receptor fucosylation by deletion of *FX* (homolog of human GDP-l-fucose synthase) or *O-fucosyltransferase (Pofut1)* has been implicated in the development of myeloid hyperplasia (Figure 2) (78). Recently, O-mucin-type glycans have been found on Notch proteins and defects in O-mucin type glycosylation are well-documented in several forms of cancer (3, 19). Defects in ppGALNAcTS (an enzyme involved in initiating O-mucin glycans) (79) and C1GalT1 (chaperones involved in elongation) (80) can lead to the truncation of Notch protein. The association of Notch proteins with ppGALNAcTs and C1GalT1 is yet to be identified. Although the functional defects of glycosyltransferases are correlated to development of cancer, further investigation is required to understand how alterations in glycosylation of Notch contribute to tumor development.

Factors that influence Notch heterodimerization can have a significant impact on receptor–ligand interactions. Interestingly, OFut1 (55, 81) and Rumi (46, 82) are proposed to bind to calcium ions to enhance rigidity and also aid in modulating the thermodynamics of the protein. It has been shown that calcium binds to the EGF 12 repeat (55, 83). Calcium binding on EGF repeats is a highly conserved phenomenon assigning a crucial role to the structure of the protein (84). Calcium binding occurs on certain amino acids on a short linker sequence, N–N–x–N–C_1_ (where N can be D/E/Q/N, x-any amino acid, and C_1_ is the first conserved Cys of the EGF) between two EGF repeats (55, 83). From our knowledge of how calcium binding can affect EGF repeats, it has been proposed that depending on the rigidity and flexibility provided, Fringe might facilitate interactions with elongated glycans or inhibit interactions with neighboring regions. Studies indicate that calcium depletion dissociates and activates heterodimeric Notch receptors (85). Reports suggest that crosstalk exists between calcium levels and Notch activity during tumorigenesis. In line with this, it has been demonstrated that calcium/calmodulin-dependent kinase II (CaMKII) regulates Notch1 activity in prostate carcinoma development (86).

### Ligand-Independent Notch-Mediated Tumorigenesis

In recent years, several reports indicate that ligand-independent Notch signaling is implicated in tumorigenesis. Several endocytic components have been associated with Notch in promoting tumor progression. In spite of this, regulatory mechanisms that initiate ligand-independent Notch signaling activity remain elusive. It is highly logical to think that such events are triggered during early stages of nascent Notch protein production in the Golgi compartment. Glycosylation is not only indispensable for protein folding and protein activity, but it has an unprecedented role in intracellular transport/localization and degradation/half-life of the protein.

Ofut1/Pofut1 has both enzymatic fucosyl activity and fucosyl-independent chaperone activity on Notch proteins (39, 87). In addition to its usual role as O-fucosyltransferase, OFut1 has been implicated in maintaining the Notch pool by recycling cell-surface Notch through endosomes and on to lysosomes in a fucose-dependent manner (39). Similarly, another study has provided evidence of the involvement of OFut1 and fucosylation in localizing Notch to the sub-apical complex/adherens junction of epithelial cells by dynamin dependent transcytosis (88). These interesting data prompt further investigation into the possible mechanisms of the process. Fringe activity follows the Ofut1 reaction on specific EGF repeats of Notch. There is evidence indicating possible glycosylation events on other sites of EGF repeats too. Therefore, Fringe activity on different EGF repeats of Notch proteins, or yet to be identified glycosylation activity, might promote cleavage of Notch that inhibits the localization of processed Notch protein to the plasma membrane, retaining it in the intracellular compartment (Figure 1B). Deregulated function of Fringe or glycosyltransferase like Ofut1 might possibly lead to aberrant Notch activity. Rumi activity has been demonstrated to be required for ligand-independent Notch activation caused by deletion of LNR repeats (47, 48). Mutations in the heterodimerization domain on the EGF repeats may impair S2 cleavage of Notch leading to either ligand-independent activation or ligand-mediated hypersensitivity. A recent report has shown a cooperation of Ofut1 chaperone activity and Rumi in Notch transport (40). In the absence of ligands, preliminary results demonstrating glycosylation-mediated Notch trafficking defects are yet to be linked to tumorigenesis.

## Perspective

Studies to date, in most contexts, demonstrate that the addition of O-glucose positively regulates Notch signaling, while updates from Shams/GXYLT suggest that the addition of O-xylose residues downregulates Notch activity in a context-specific manner. It is proposed that this regulation, by changing the distribution of forms and length of sugar residues, offers a novel paradigm to modulate Notch signaling (48). Report suggests that the addition of Xylose to isolated Ser or Thr residues initiates Glycosaminoglycans (GAG) synthesis (89). As several studies have implicated aberrant GAG synthesis and GAG-conjugated proteins to tumor development and metastasis (90–92), it is highly intriguing to understand the significance of GAGs during Notch-induced tumorigenesis. Current therapeutic developments depend mostly on either modulating ligand–receptor interactions or the proteolytic cleavage of the receptor (93). Recent studies indicate that glycosylating proteins are important auxiliary proteins that modulate Notch activity and could therefore also be potential targets for future therapeutics. Glycan profiling of the modified glycans on Notch proteins may provide a better picture to understand the dramatic “glycome shift” that takes place during tumorigenesis and metastasis.

It is clear from the numerous studies highlighted here that Notch regulation is extremely complex and context dependent. For example, Notch can signal in a ligand-dependent or -independent manner, there are multiple Notch ligands, and the plethora of glycosyl modifications discussed in this review provide a further level of complexity. It is also clear how little we understand about how this regulation affects Notch’s ultimate function, namely, the regulation of gene expression. How does the nature of the ligand (or lack of ligand) affect the combination of genes whose expression will be upregulated or downregulated due to Notch signaling? Do individual or combinations of glycosyl modifications affect Notch’s ability to engage with other DNA-binding proteins and regulate the expression of specific genes? Notch-mediated gene regulation controls multiple cell differentiation processes both during development and adult life, and the complexity of Notch regulation will likely provide the necessary specificity that is required to generate the correct response to Notch signaling in different contexts. Our lack of understanding is tangible and unless we address these questions we cannot begin to understand how Notch regulation leads to specificity of response or the mechanisms by which Notch deregulation can lead to either an oncogenic or tumor-suppressive effect.

## Author Contributions

SP, AC, UN, and MG wrote and revised the manuscript.

## Conflict of Interest Statement

The authors declare that the research was conducted in the absence of any commercial or financial relationships that could be construed as a potential conflict of interest.
